# Intermittent Swelling of the Parotid Glands

**Published:** 1913-09

**Authors:** C. W. J. Brasher

**Affiliations:** Hon. Medical Registrar, Bristol Royal Infirmary.


### INTERMITTENT SWELLING OF THE PAROTID GLANDS.

BY

C. W. J. BRASHER, M.D., Ch.B. Bristol,

*Hon. Medical Registrar, Bristol Royal Infirmary.*

---

In the *British Medical Journal* of August 10th, 1912 (p. 328), there is an interesting article on this subject.

Seven cases have been recorded by Goebel, Remouchamp, Kussmaul, Lange and Lüders. Only one patient was a woman; of the six men two had had syphilis, but although both received energetic anti-syphilitic treatment, the recurrent inflammation of the parotid glands continued.

In Kussmaul's case the periodical swelling was followed by escape of fibrinous plugs from Stenson's ducts, accompanied by a free flow of saliva.

In Remouchamp's case (a man of 85, who had suffered from this disease for fifty-nine years) chemical and microscopical examination of the saliva showed that it was alkaline, rich in mucin and ptyalin, and that it contained pus cells, large nucleated leucocytes, fragments of leucocytes, streptococci and short gram-staining rods, and a blood-count revealed a marked degree of leucocytosis. This appears to be the only case in which a bacteriological examination of the saliva has been made.

Lüders considers that the disease (in his two cases) was due to " chills," and the writer of the article thinks that " the above cases were probably due to catarrh of the mucous membrane lining the ducts of the glands."

Drugs appear to have had little effect upon the swelling, and the treatment consisted chiefly of fomentations during an attack and subsequent massage of the glands.

In one case only (that of a woman aged 39) was there any abscess formation ; this occurred seven years after the first attack, and the abscess was opened and drained.

In the following case iron, strychnine, quinine and belladonna were given at intervals for a considerable time, and during the attacks fomentations, ichthyol-glycerine and belladonna liniment were applied, but the results were disappointing. Massage was never employed, as the glands were always too tender, even when of normal size.

It is interesting to note that the patient had suffered for many years from chronic nasal catarrh with frequent exacerbations, and had had one or two severe attacks of acute bronchitis before the recurrent parotid inflammation commenced.

On October 24th, 1901, I saw Miss Mary G., aged 26, head mistress of a public elementary school. She was suffering from painful swelling of both parotid glands with salivation, " cold shivers " and general malaise. The temperature was 100° and the pulse-rate 88. I told the patient that she had an attack of " mumps," and inquired if there were any cases in her school. Her reply was rather surprising, and, at the time, unconvincing : " This is not infectious ; I have had it several times before." And she said further that she had been treated for the same disease on several occasions by her former medical attendant, who eventually sent her to a laryngologist for treatment of the chronic nasal catarrh.

I was still incredulous, and insisted upon isolation, as there were two small children in the house. The swelling subsided in four or five days, as in an ordinary attack of mumps ; there was no metastatic inflammation, and no other case occurred in the house.

The patient was not seen again until November 5th, 1907, an interval of more than six years, when she had a similar attack affecting both parotids, and she then told me that she had had several attacks in the interval, the swellings being sometimes unilateral, but usually both glands were affected, though it occasionally happened that, as in ordinary " mumps," the inflammation of one gland preceded that of the other by a few days.

Another attack occurred on March 31st, 1908, *i.e.* in less than five months, and there were recurrences in the following December and again on March 4th, 1909. On July 19th there was another recurrence, followed by an interval of eleven months. The next attack occurred on June 23rd, 1910 ; this was more severe and lasted for a week. The next attack occurred in seven months, *i.e.* January 23rd, 1911 ; and the last recurrence commenced in ten months on November 24th, 1911.

Miss G. informed me that she could not tell how many slight attacks occurred at intervals of a few months, and she only sent for me when the swelling was so severe and painful that she could not go to school ; but I attended her in no less than nine attacks, each one reproducing the ordinary symptoms of uncomplicated "mumps." At no time was there any mastitis ; menstruation has always been painless and quite regular ; the teeth and digestive organs are quite healthy. Every attack was followed by lassitude, neuralgic pain in the face, and much tenderness of the parotid glands, persisting after all obvious swelling had subsided. During and after the attacks the patient was treated with iron, quinine, strychnine and belladonna, and on one or two occasions sodium salicylate was prescribed. Hot fomentations and belladonna liniment were used, and on several occasions ichthyol-glycerine was applied. The periodical recurrence, uninfluenced by ordinary treatment, suggested a chronic bacterial infection, and after discussing the case with Professor Walker Hall, I asked the patient to send for me when an attack was coming on. She came to see me on June 23rd, 1911, when an attack was at its height, the left gland being more swollen than the right. The buccal mucous membrane around the orifice of Stenson's duct on the left side was well washed with a 20 per cent. solution of hydrogen peroxide, and a small sterilised swab on a fine probe was introduced into the patulous orifice. Saliva was flowing freely at the time, and it proved to be quite sterile.

The patient was told to send earlier next time, and she did so on November 24th, 1911, when another attack was commencing. The same technique was carried out, and a swab was introduced into the left duct. On this occasion we were more successful. The culture yielded a pure growth of a gram-negative micrococcus, morphologically identical with micrococcus catarrhalis. Professor Walker Hall prepared a vaccine, and the patient received nineteen injections in increasing doses. She suffered from considerable malaise during the course of injections and complained of much "tingling pain" in the parotid glands.

She had a severe attack of acute bronchitis in April, 1912, but remained free from parotid inflammation for more than a year—a longer period than at any time during the last eleven years. The after-history of the case, however, is very disappointing.

Miss G. went to Cheshire to spend her Christmas holidays, and whilst there had another attack, as severe as that in November, 1911. Dr. Robertson, of Dukinfield, suggested that it might be a form of rheumatism, and prescribed aspirin in 15-grain doses. " This," she wrote, " relieved the pain far more speedily than anything I had previously had," but naturally she was very depressed by the recurrence. The patient suffered from constant lassitude and depression, but had no further attack until March 11th, 1913, when both parotid glands suddenly swelled simultaneously and the inflammation persisted for eleven days; the temperature never rose above 102 deg., but there was great dysphagia, loss of appetite, and general malaise. Aspirin in 15-grain doses was given from the commencement of the attack, but after a few days it appeared to cause gastritis, so natural sodium salicylate in similar doses was prescribed; neither drug, however, appeared to give any relief. When the swellings subsided an intensely tender nodule as large as a hazel nut was felt over the posterior border of the right masseter; it appeared to be a retention-cyst. On May 9th it had contracted to the size of a large pea, and was still very tender.

This case has followed the course of the seven previously recorded, in that all treatment has been simply palliative, and nothing that has been tried has prevented frequent recurrences in every case. In regard to the vaccine treatment, the question arises, Did the vaccine confer a temporary passive immunity which protected the patient for a year, or was the absence of an attack during that period a mere coincidence? It will be remembered that the general experience of micrococcus catarrhalis vaccines suggests that the resultant period of immunity is comparatively short, and does not usually last for more than eighteen months or two years. The failure of the vaccine would have been less remarkable had the culture shown a mixed infection, as in Remouchamp's case, quoted above.

It may well be that these cases are not so rare as one might suppose, in view of the fact that this appears to be only the eighth recorded case, but it is probable that many more have escaped attention; and if anything can be done to relieve a very painful and distressing condition, it is necessary to collect as many cases as possible with details of their treatment.

